# Renal resistive index and neonatal sequential organ failure assessment score as acute kidney injury risk predictors in preterm neonates with late-onset neonatal sepsis

**DOI:** 10.3389/fneph.2026.1734812

**Published:** 2026-05-25

**Authors:** Abed Ricky H. Sitompul, Tetty Yuniati, Fiva Aprilia Kadi, Aris Primadi, Dwi Prasetyo, Ahmedz Widiasta, Ekawati L. Haksari, Harry G. Nugraha, Antony A. Adibrata

**Affiliations:** 1 Neonatology Sub-Specialist Program, Department of Child Health, Faculty of Medicine, Padjadjaran University, Bandung, Indonesia; 2 Department of Child Health, Faculty of Medicine, Padjadjaran University/Dr. Hasan Sadikin General Hospital, Bandung, Indonesia; 3 Department of Child Health, Faculty of Medicine, Gadjah Mada University, Sardjito General Hospital, Yogyakarta, Indonesia; 4 Department of Radiology, Faculty of Medicine, Padjadjaran University/Dr. Hasan Sadikin General Hospital, Bandung, Indonesia; 5 Faculty of Medicine, Padjadjaran University, Bandung, Indonesia

**Keywords:** acute kidney injury, kidney assessment, neonatal sepsis, preterm neonates, risk stratification scores

## Abstract

**Aim:**

Neonatal acute kidney injury (AKI) is an independent predictor of mortality with serum creatinine (sCr) remaining the gold standard. However, by the time sCr increases, the kidney may already have lost part of its function. Late-onset neonatal sepsis (LONS) is a common trigger of AKI through inflammation and renal hypoperfusion. The renal resistive index (RRI) is a Doppler-derived marker of renal perfusion, and the neonatal sequential organ failure assessment (nSOFA) score reflects systemic organ dysfunction that may accompany sepsis-associated AKI. This study aimed to compare the diagnostic performance of the RRI and nSOFA scores for predicting AKI risk in preterm neonates with probable LONS, using the STARZ (Sethi Tibrewal Agarwal Raina waZir) score for risk stratification.

**Methods:**

This is a single-center prospective study that observed all neonates admitted to the neonatal intensive care unit (NICU) with probable LONS from July to August 2025 defined using the sepsis prediction score. Clinical and laboratory data included the STARZ score, RRI, and the nSOFA score, which were obtained at birth and a few times after the diagnosis of probable LONS based on the sepsis prediction score.

**Results:**

A total of 36 preterm neonates were included. Median RRI and STARZ scores increased during the course of LONS, while nSOFA scores significantly differed between high- and low-risk AKI groups on multiple time points. Birth weight <1, 357 g and congenital heart disease were significant risk factors. Receiver operating characteristic (ROC) analysis showed birth weight <1, 357 g [area under the curve (AUC), 0.708; sensitivity, 72.22%; and specificity, 77.78% (*p* = 0.026)] and nSOFA score >3 on day 7 [AUC, 0.724; sensitivity, 61.11%; and specificity, 77.78% (*p* = 0.01)]. Multivariate analysis confirmed nSOFA score >3 on day 7 as a strong independent predictor of high-risk AKI [odds ratio (OR), 10.01; 95% confidence interval (CI), 1.18–85.13].

**Conclusions:**

Serial nSOFA monitoring in preterm neonates with LONS may provide practical and accessible risk stratification for AKI and could support earlier identification of infants at high risk.

## 1 Introduction

Neonatal acute kidney injury (AKI) is a common issue encountered in neonatal intensive care units (NICUs) and recognized as an independent predictor of mortality (1, 2). Conditions such as prematurity, exposure to nephrotoxic drugs, sepsis, congenital heart disease (CHD), hypoxic–ischemic encephalopathy (HIE), and necrotizing enterocolitis (NEC) place neonates at high risk for developing AKI (3, 4). The gold standard for diagnosing neonatal AKI remains serum creatinine (sCr) measurement, which primarily reflects kidney function rather than direct kidney injury (1). By the time sCr increases, kidney function loss has already occurred between 20% and 50%, resulting in delayed detection of neonatal AKI and subsequently increased neonatal morbidity and mortality (1).

Various methods and biomarkers have been proposed to prompt the detection of AKI. Novel biomarkers, including cystatin C, neutrophil gelatinase-associated lipocalin (NGAL), interleukin-18 (IL-18), kidney injury molecule-1 (KIM-1), osteopontin (OPN), and beta-2 microglobulin (B2mG), have demonstrated high diagnostic accuracy in identifying AKI at an early stage (7–13). However, these biomarkers have been primarily evaluated in high-risk populations with a high incidence of AKI, and are yet to be standardized or widely applied across all neonatal populations due to lack of consensus cutoff values. In addition to biomarker-based approaches, clinical monitoring programs have also been implemented, such as baby NINJA (Nephrotoxic Injury Negated by Just-in-Time Action), which began in 2019 by monitoring sCr periodically. The program manages to detect and reduce the incidence of AKI in neonates; therefore, the AKI risk stratification score is expected to become a standard operating procedure for adjusting therapy in NICU patients to prevent AKI (5, 6). Nonetheless, because of the challenges in diagnosing neonatal AKI based on biomarkers alone, another neonatal AKI risk stratification score was developed: the STARZ (Sethi Tibrewal Agarwal Raina waZir) score was established as a tool for early risk stratification in neonatal AKI (14). However, studies validating the STARZ score across various neonatal populations and clinical conditions remain limited (14, 15).

Late-onset neonatal sepsis (LONS) was defined as sepsis occurring after 72 h of life, and it is associated with increased mortality, neurodevelopmental impairment, and higher hospital care costs (16). Neonatal sepsis can cause direct renal injury through cytokine release and oxidative stress leading to tubular apoptosis and microthrombotic damage. Indirectly, sepsis induces renal hypoperfusion, triggering ischemic kidney injury (4). Renal hypoperfusion can be assessed using Doppler ultrasonography of the renal arteries, with the renal resistive index (RRI) serving as a parameter reflecting renal perfusion essential for glomerular filtration rate (GFR) (7). Early detection of RRI changes may indicate GFR alterations before significant sCr changes occur. However, limited research exists utilizing RRI alone to detect neonatal AKI. Pediatric and adult studies have reported that RRI cutoff values below 0.68 have a sensitivity of 83% and a specificity of 82% for AKI detection (8).

Beyond the STARZ score and RRI, neonatal AKI risk assessment can be enhanced by using the neonatal sequential organ failure assessment (nSOFA) score (17). Although nSOFA has been validated for severity assessment in neonatal sepsis and other critical conditions, evidence regarding its utility for AKI risk stratification during LONS remains limited. Similarly, while RRI has been explored as a non-invasive indicator of renal perfusion, its diagnostic performance for neonatal AKI is inconsistent across settings. Therefore, this study aimed to compare the diagnostic performance of RRI and serial nSOFA scores for predicting AKI risk in preterm neonates with probable LONS, using STARZ-based risk stratification as the reference comparator.

## 2 Materials and methods

### 2.1 Patients

This was a single-center prospective study that included 36 preterm neonates with probable LONS from July to August 2025. The inclusion criteria were all neonates with a gestation age of 28–36 weeks who met the sepsis prediction score (SPS) threshold for probable late-onset sepsis (≥3 of 8) (18). LONS was defined as sepsis occurring after 72 h of life.

Neonates with major congenital anomalies expected to substantially affect survival or organ function were excluded, including congenital anomalies of the kidney and urinary tract (CAKUT) (e.g., renal agenesis, renal dysplasia, or obstructive uropathy). CHD was not an exclusion criterion and was analyzed as a comorbidity, including patent ductus arteriosus (PDA), when clinically diagnosed or considered hemodynamically significant by the treating team.

### 2.2 Data collection

Study participants were observed for 7 days after being diagnosed with probable LONS. Demographic and clinical variables were included, including sex, gestational age, birth weight, delivery method, antenatal corticosteroid, age at LONS diagnosis, comorbidities, duration of mechanical ventilation, duration of vasopressor/inotropic support, and AKI incidence. Clinical and laboratory parameters included sCr, STARZ score, RRI, and nSOFA score. These parameters were collected at baseline (day 0: time of LONS diagnosis) and subsequently on day 1, day 3, and day 7 after diagnosis.

RRI measurement was performed by a pediatric radiologist using renal Doppler ultrasonography. Doppler sampling was obtained from intrarenal arteries (interlobar or arcuate arteries) with an appropriate insonation angle, and at least three consecutive, technically adequate waveforms were recorded. RRI was calculated as (peak systolic velocity − end diastolic velocity)/peak systolic velocity. Measurements were performed at predefined time points (days 0, 1, 3, and 7 after LONS diagnosis), and the value used for analysis was the average of repeated measurements recorded at each time point.

AKI in this study was defined using neonatal modified KDIGO criteria, based on serial changes in sCr (and urine output criteria when reliably available in the medical record). In addition, AKI risk stratification was assessed using the STARZ score, where a score ≥31.5 indicates high risk for AKI and a score <31.5 indicates low risk (15). STARZ risk grouping was used as the primary reference comparator to evaluate bedside predictors in this small cohort; limited external validation of STARZ is acknowledged as a limitation.

### 2.3 Ethics

The study was conducted in accordance with the Declaration of Helsinki and approved by the Research Ethical Committee of Hasan Sadikin General Hospital in Bandung, Indonesia (approval number DP.04.03/D.XIV.6.5/174/2025). Written informed consent was obtained from all the participant’s parents or guardian prior to their inclusion. Each of them received an information sheet outlining the objectives, procedures, and benefits of the study. Participation was entirely voluntary, with no compensation provided.

### 2.4 Statistical analysis

All statistical analyses were performed using SPSS version 29.0. Categorical variables are presented as *n* (%). Continuous variables are presented as mean ± standard deviation (SD) for normally distributed data or median (range or IQR) for non-normally distributed data, as appropriate. Normality was assessed using the Kolmogorov–Smirnov test.

Comparisons between high-risk and low-risk AKI groups (STARZ ≥31.5 vs. <31.5) were performed using the chi-square test or Fisher’s exact test for categorical variables and the independent *t*-test or Mann–Whitney *U* test for continuous variables, as appropriate. For analyses involving more than two groups, analysis of variance (ANOVA) or Kruskal–Wallis testing was used depending on distribution. Variables showing significant differences (*p* < 0.05) were evaluated using receiver operating characteristic (ROC) curve analysis to identify cutoff values for predicting high-risk AKI. The association between derived cutoffs and AKI risk category was assessed using chi-square/Fisher’s exact testing. Two primary predictors (RRI and nSOFA cutoffs), together with significant clinical covariates, were entered into multivariable logistic regression to identify independent predictors of high-risk AKI. A two-sided *p*-value <0.05 was considered statistically significant.

## 3 Results

### 3.1 Patient baseline characteristics

**Figure 1:**
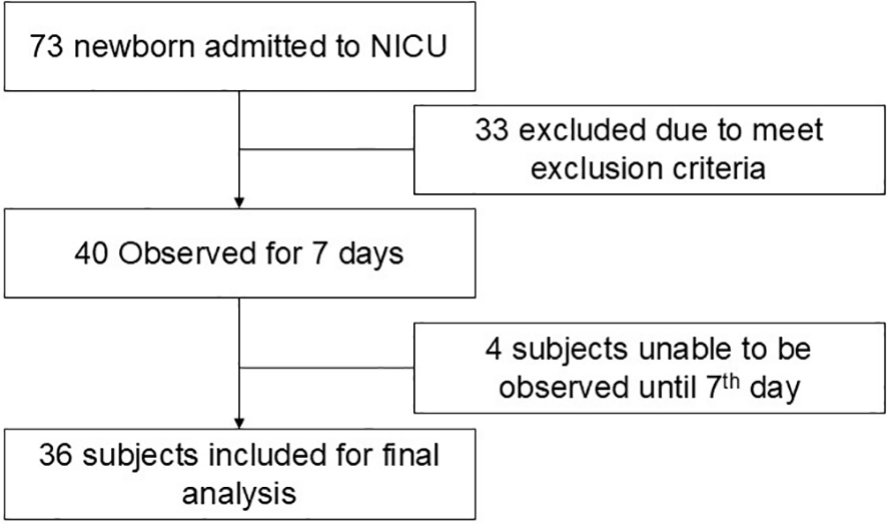
Patient selection process.

During the study period, 73 neonates were admitted to the NICU. Forty met eligibility criteria; 4 were excluded due to incomplete 7-day follow-up, leaving 36 neonates included in the final analysis (**Figure 1**). Records were taken for all research subjects, including characteristic data such as gender; birth weight; gestational age; gestational age appropriateness [small for gestational age (SGA)]; type of delivery; antenatal steroid administration; age at diagnosis of probable LONS; comorbidities including NEC, CHD, and IVH; duration of ventilator use; use of inotropics/vasopressors; incidence of AKI; and use of caffeine. Blood culture results were not consistently available in the medical records for the study period; therefore, we did not analyze culture positivity rates in this cohort.

Baseline characteristics of study participants are presented in **Table 1**. There was no significant difference in gender distribution, with 19 male (52.8%) and 17 female (47.2%) patients having an average birth weight of 1, 436.6 g (SD 479.3). Most babies had a birth weight between 1, 500 and 2, 499 g (47.2%). The type of delivery was predominantly cesarean delivery, *n* = 29 cases (80.6%), with most mothers receiving antenatal corticosteroids, *n* = 27 cases (75.0%). The mean age at diagnosis of LONS was 153.6 h (SD 92.5). Some infants had comorbidities, namely, NEC recorded in 11 neonates (30.6%), CHD in 15 subjects (41.7%), and IVH in 10 subjects (27.8%). The majority of the subjects in the study required mechanical ventilation (88.9%). The median duration of ventilator use increased over time, with a median of 264 h on the 7th day of observation. sCr levels tended not to increase during LONS.

**Table 1:** Baseline characteristics.

Variable	*n* (%)/Mean (SD)
Sex	
• Male • Female	19 (52.8) 17 (47.2)
Birth weight (g), Mean (SD) • <1, 000 • 1, 000–1499 • 1, 500–2, 499 • >2, 500	1, 436.6 (479.3) 8 (22.2) 10 (27.8) 17 (47.2) 1 (2.8)
Gestation age (weeks)	31.5 (2.3)
Small-for-gestation age (SGA)	
• Yes • No	11 (30.6) 25 (69.4)
Delivery method	
• Vaginal delivery • Cesarean delivery	7 (19.4) 29 (80.6)
Antenatal corticosteroid	
• Yes • No	27 (75.0) 9 (25.0)
Age at probable LONS diagnosis (h)	153.6 (92.5)
Comorbid • NEC • CHD • IVH	11 (30.6) 15 (41.7) 10 (27.8)
Ventilator usage	32 (88.9)

LONS, late-onset neonatal sepsis; NEC, necrotizing enterocolitis; CHD, congenital heart disease; PDA, patent ductus arteriosus; IVH, intraventricular hemorrhage; SD, standard deviation.

### 3.2 Risk of acute kidney injury

**Table 2** shows clinical characteristics over 7 days of observation. Over the 7-day observation period, clinical support needs generally increased such as ventilator duration and vasoactive/inotropic usage. sCr decreased from day 0 to day 1 and then remained fairly stable. KDIGO-defined AKI was uncommon but appeared after baseline (0 cases on day 0, 1 on day 1, 2 on day 3, and 2 on day 7). During follow-up, median RRI, nSOFA, and STARZ values increased, and the proportion classified as high risk (STARZ ≥31.5) increased from 33.3% on day 0 to 58.3% on day 1 and remained approximately half of the cohort on days 3 and 7.

**Table 2:** Time-varying variables on day 0, day 1, day 3, and day 7.

Variable	Day of observation			
	0 *n* (%)	1 *n* (%)	3 *n* (%)	7 *n* (%)
Ventilator duration (h) Median (IQR)	0 (-)	96 (0–142.5)	168 (72–214.5)	264 (168–312)
Serum creatinine Median (IQR)	0.69 (0.59–0.83)	0.55 (0.45–0.70)	0.56 (0.41–0.71)	0.56 (0.45–0.71)
Inotropic/vasopressor Mean (SD)	10 (27.8)	17 (47.2)	17(47.2)	18 (50)
Caffeine Mean (SD)	26 (72.2)	27 (75.0)	28 (77.8)	28 (77.8)
Acute kidney Injury incidence	0	1	2	2
Renal resistive index Median (IQR)	0.78 (0.74–0.88)	0.83 (0.78–0.90)	0.86 (0.80–0.91)	0.87 (0.79–0.93)
nSOFA score Median (IQR)	0 (0–2)	3 (2–3.75)	3 (0–5.75)	3 (1.25–6.0)
STARZ Median (IQR)	25.5 (17–37)	38 (18.25–44)	33.5 (18.25–43.75)	32 (17–46.25)
STARZ category: *n* (%) • ≥31.5 (High risk) • <31.5 (Low risk)	12 (33.3) 24 (66.7)	21 (58.3) 15 (41.7)	19 (52.8) 17 (47.2)	18 (50.0) 18 (50.0)

LONS, late-onset neonatal sepsis; NEC, necrotizing enterocolitis; CHD, congenital heart disease; PDA, patent ductus arteriosus; IVH, intraventricular hemorrhage; RRI, renal resistive index; nSOFA, neonatal sequential organ failure assessment; STARZ, STARZ neonatal AKI risk score; sCr, serum creatinine; IQR, interquartile range; SD, standard deviation.

**Table 3** shows a comparison of RRI and the nSOFA score for risk of AKI based on STARZ high-risk and low-risk groups. RRI did not differ meaningfully at any time point, while nSOFA was consistently higher in the high-risk group on day 0 (median 2 vs. 0; *p* = 0.049), day 1 (median 3 vs. 2; *p* = 0.007), and day 7 (median 5 vs. 2; *p* = 0.020), while day 3 was not significant (*p* = 0.129). However, **Table 4** shows that there were consistent differences in characteristics between high-risk and low-risk AKI in terms of birth weight and CHD, with *p*-values of 0.025 and 0.002. Ventilator use was significantly meaningful on days 1 and 3, but not significant on days 0 and 7.

**Table 3:** Risk of acute kidney injury based on observation days.

Variable	Acute kidney injury		*p*-value
	High risk	Low risk	
Renal resistive index			
• Day 0, mean (SD)	0.817 (0.092)	0.787 (0.115)	0.426^a^
• Day 1, mean (SD)	0.857 (0.094)	0.815 (0.068)	0.158^a^
• Day 3, median (IQR)	0.86 (0.76–1.00)	0.85 (0.77–0.99)	0.975^b^
• Day 7, mean (SD)	0.872 (0.089)	0.854 (0.095)	0.565^a^
nSOFA score:			
• Day 0, median (IQR)	2 (0–4)	0 (0–3)	0.049^b^
• Day 1, median (IQR)	3 (0–10)	2 (0–5)	0.007^b^
• Day 3, median (IQR)	4 (0–12)	2 (0–8)	0.129^b^
• Day 7, median (IQR)	5 (0–14)	2 (0–14)	0.020^b^

a Analyses using independent *t*-test; ^b^analyses using the Mann–Whitney *U* test.

**Table 4:** Characteristics among high risk and low risk of acute kidney injury based on days of observations.

Characteristic	AKI high risk Day 0 (*n* = 12)	AKI low risk Day- 0 (*n* = 24)	*p*-value*	AKI high risk Day 1 (*n* = 21)	AKI low risk Day 1 (*n* = 15)	*p*-value*	AKI high risk Day 3 (*n* = 19)	AKI low risk Day 3 (*n* = 17)	*p*-value*	AKI high risk Day 7 (*n* = 18)	AKI low risk Day 7 (*n* = 18)	*p*-value*
Sex												
• Male • Female	6 6	13 11	0.813	11 10	8 7	0.955	11 8	8 9	0.516	12 6	7 11	0.095
Birth weight (g)												
• <1, 000 • 1, 000–1, 499 • 1, 500–2, 499 • >2, 500	4 6 1 1	4 4 16 0	0.008	8 8 4 1	0 2 13 0	0.001	6 9 3 1	2 1 14 0	0.001	6 7 4 1	2 3 13 0	0.025
Small-for-gestation age												
• Yes • No	4 8	7 17	0.798	7 14	4 11	0.669	5 14	6 11	0.559	5 13	6 12	0.717
Delivery method												
• Vaginal delivery • Cesarean delivery	3 9	4 20	0.551	5 16	2 13	0.434	5 14	2 15	0.271	4 14	3 15	0.674
Antenatal corticosteroid												
• Yes • No	9 3	18 6	1.00	17 4	10 5	0.329	16 3	11 6	0.177	15 3	12 6	0.248
Comorbid												
• NEC • CHD • IVH	3 9 1	8 6 9	0.609 0.004 0.066	8 13 6	3 2 4	0.245 0.004 0.990	8 13 5	3 2 5	0.112 0.001 0.836	7 12 5	4 3 5	0.278 0.002 1.00
Ventilator usage												
• Yes • No	12 0	20 4	0.134	21 0	11 4	0.012	19 0	13 4	0.025	17 1	15 3	0.289

AKI, acute kidney injury; NEC, necrotizing enterocolitis; CHD, congenital heart disease; IVH, intraventricular hemorrhage. *Chi-square or Fisher’s exact test was used for categorical data; the Mann–Whitney *U* test was used for birth weight and age.

**Figure 2:**
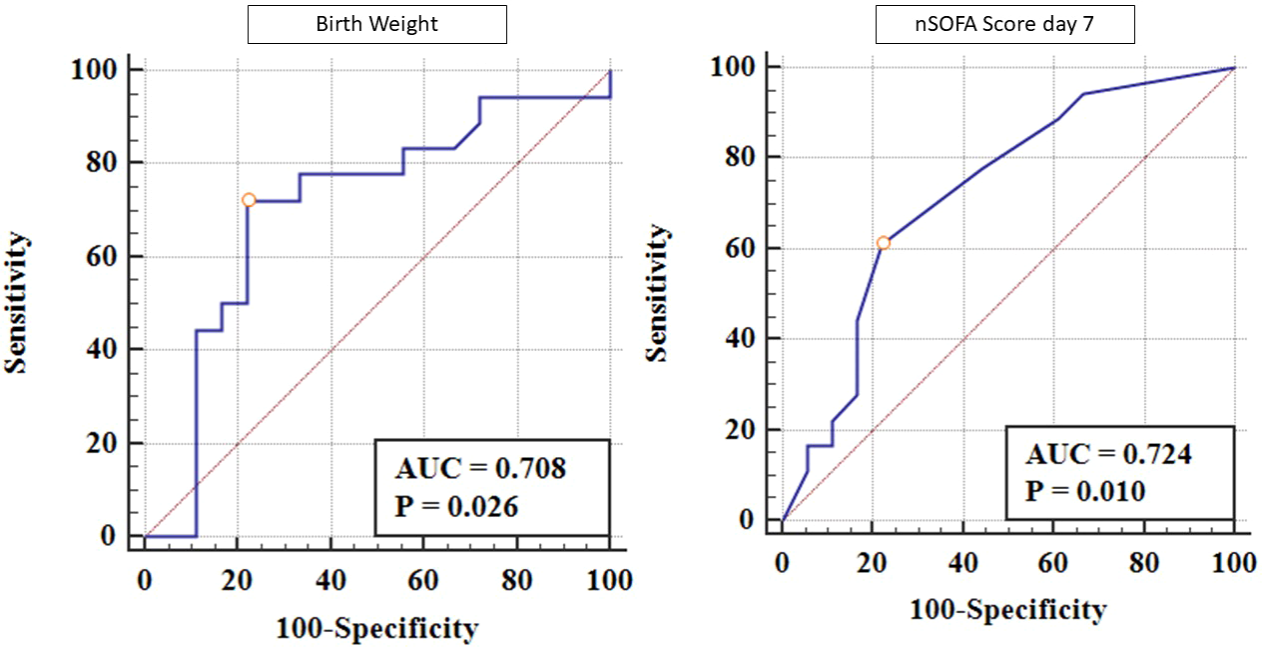
The ROC curve of birth weight and nSOFA score for the prediction of high-risk kidney injury in late-onset neonatal sepsis. The red circle marks the optimal cutoff based on the Youden index: birth weight (AUC, 0.708; sensitivity, 72.22%; specificity, 77.78%); nSOFA day 7 (AUC, 0.724; sensitivity, 61.11%; specificity, 77.78%).

**Table 5** and **Figure 2** show an ROC analysis of high-risk AKI on five variables that were significant in the bivariate test with a meaningful cutoff obtained in the birth weight <1, 357 g and nSOFA >3 day 7. Birth weight ≤1, 357 g had an area under the receiver operating characteristic curve (AUROC) of 0.708, resulting in a sensitivity of 72.22% and a specificity of 77.78% (*p* = 0.026), and nSOFA >3 on day 7 had an AUROC of 0.724, resulting in a sensitivity of 61.11% and a specificity of 77.78% (*p* = 0.01). Cutoff value was determined by using the Youden index. In unadjusted analyses, birth weight ≤1, 357 g, nSOFA day 1 >5, and nSOFA day 7 >3 were significantly associated with high-risk status (**Table 6**). However, after adjustment in multivariable logistic regression, only nSOFA >3 on day 7 remained an independent predictor [adjusted odds ratio (OR), 10.01; 95% confidence interval (CI), 1.18–85.13; *p* = 0.035] (**Table 7**).

**Table 5:** Cutoff value for high risk of acute kidney injury.

Variable	Cutoff value	AUROC (95% CI)	*p*-value	Sensitivity (%)	Specificity (%)	PPV (%)	NPV (%)	LR+	LR−
Birth weight (g)	≤1, 357	0.708 (0.533–0.847)	0.026	72.22	77.78	76.5	73.7	3.25	0.36
RRI day 1	>0.8	0.653 (0.476–0.803)	0.104	50.0	60	60	72.7	1.67	0.33
nSOFA day 0	>1	0.637 (0.461–0.791)	0.079	44.44	83.33	72.7	60	2.67	0.67
nSOFA day 1	>5	0.650 (0.473–0.801)	0.097	27.78	100	100	58.1	2.5	0.72
nSOFA day 7	>3	0.724 (0.550–0.859)	0.010	61.11	77.78	73.3	66.7	2.75	0.5

AUROC, area under the receiver operating characteristic curve; PPV, positive predictive value; NPV, negative predictive value; LR+, positive likelihood ratio; LR−, negative likelihood ratio; RRI, renal resistive index.

**Table 6:** Correlation of cutoff value with high risk of acute kidney injury.

Variable	Cutoff value	Acute kidney injury		*p*-value*	RR (95% CI)
		High risk	Low risk		
Birthweight (g)	≤1, 357 >1, 357	13 5	4 14	0.003	2.91 (1.31–6.45)
RRI day 1	>0.8 ≤0.8	15 3	10 8	0.070	2.20 (0.80–6.08)
nSOFA day 0	>1 ≤1	8 10	3 15	0.070	1.82 (1.00–3.32)
nSOFA day 1	>5 ≤5	5 13	0 18	0.045	2.38 (1.58–3.61)
nSOFA day 7	>3 ≤3	11 7	4 14	0.018	2.20 (1.12–4.33)

*Chi-square or Fisher’s exact test. RR (95% CI): Relative risk (95% confidence interval).

**Table 7:** Multivariable analysis of AKI high risk according to multiple logistic regression.

Variable	Coef *B*	SE (*B*)	*p*-value	Adj OR (95% CI)
CHD comorbid Birth weight (≤1, 357 g) RRI day 1 (>0.8) nSOFA day 0 (>1) nSOFA day 7 (>3)	1.726 1.673 2.733 2.106 2.304	1.044 1.047 1.541 1.302 1.092	0.098 0.110 0.076 0.106 0.035	5.62 (0.73–43.43) 5.33 (0.68–41.45) 15.38 (0.75–315.30) 8.22 (0.64–105.39) 10.01 (1.18–85.13)

Adj OR (95% CI): adjusted odds ratio (95% confidence interval). Accuracy = 83.3%; *R*^2^ (Nagelkerke) = 0.627.

## 4 Discussion

Currently, diagnosing AKI is challenging due to reliability in sCr that occurred 48–72 h after the damage occurred. Alternative scoring was proposed for measuring AKI stratification and the STARZ score, to increase awareness of AKI events before they occur. The STARZ score has a sensitivity strength of 92.8% and a specificity of 87.4% (14). Another measurement such as RRI, using ultrasound as bedside real-time monitoring, provides a new paradigm of renal Doppler in the detection of AKI (19). The nSOFA score has been used in several studies to assess the critical condition of organs that are starting to be widely used in the detection of AKI. A total of 36 subjects born with a gestational age of 28–36 weeks who experienced LONS were included in this study. The proportion of genders in this study was relatively balanced, with male at 52.8% and female at 47.2%. Askenazi et al., in a prospective cohort study, found an increased risk of AKI in male compared to female patients (20). However, a systematic review did not identify gender as a significant risk factor for risk factors for AKI in preterm infants (21). Sethi et al., in a STARZ study in 2022, also proved that sex was not significance as a risk factor for AKI in neonates (14). Average birth weight in this study was 1, 436 g ( ± 479 g), which indicates that most of the subjects in this study fall into the categories of LBW and VLBW. Chirico et al. stated that the number of nephrons is positively correlated with birth weight, and those with low birth weight have structurally and functionally reduced nephron masses, making them more susceptible to AKI (22).

The proportion of SGA neonates was 30.6%, with bivariate analysis showing no significant differences between the high- and low-risk AKI groups (*p* = 0.717). SGA neonates have a higher risk of developing AKI due to the low number of nephrons from birth and increased susceptibility of the kidneys to hemodynamic and inflammatory stresses. A case–control study by El-Sharkawy et al. showed an increased risk of AKI in the SGA population of preterm neonates compared to appropriate for gestational age (AGA), but on the other hand, the AWAKEN study showed no significant difference in the incidence of AKI among populations (23, 24). Although SGA in this study is not statistically significant, previous literature suggest that those neonates need to be watched out for as a group with a higher risk of AKI.

Most of the subjects were delivered by cesarean delivery (80.6%). In the analysis, there was no significant difference in the type of delivery to the risk of AKI (*p* = 0.443). These results are consistent with those of a systematic review and meta-analysis by Moraes et al., which show that cesarean delivery is not an independent risk factor for AKI. Cesarean delivery appeared as a factor in univariate analysis, but the effect disappeared after adjusting for gestational age, birth weight, sepsis, and mechanical ventilation (21).

In this study, 75% of subjects received antenatal corticosteroids. There was no significant difference between the high- and low-risk AKI groups (*p* = 0.389), in line with a prospective study by Pasini et al., which stated that there was no significant difference (*p* = 0.27) in the VLBW group that received antenatal corticosteroids (25). However, Utsun et al. stated that completion of antenatal administration of corticosteroids is associated with a significant reduction in the incidence of AKI (OR, 0.41; 95% CI, 0.20–0.83) and medium-to-severe AKI (OR, 0.205; 95% CI, 0.075–0.563) compared to without antenatal administration of corticosteroids. The protective mechanism is thought to be through hemodynamic maturation, improvement of lung function, and reduction of ventilation and endocrine needs (26).

The comorbidities recorded included NEC (30.6%), CHD (41.7%), and IVH (27.8%). Bivariate analysis showed that CHD was more prevalent in the high-risk AKI group and the results were statistically significant (*p* = 0.002). The comorbidity of NEC was not significant (*p* = 0.278), and the IVH was the same in both groups. The significance of CHD confirms the important role of congenital heart defects as a risk factor for AKI. Physiologically, hemodynamic shunts in CHD especially with large PDA or other significant defects lead to chronic renal hypoperfusion and increase the risk of kidney damage due to exposure of sepsis or aggressive therapy. The AWAKEN multicenter study stated that cardiovascular abnormalities were consistently associated with the incidence of AKI (*p* < 0.0001) (23). A cohort by Shalaby et al. stated that PDA increased the risk of AKI (RR, 1.2; 95% CI, 9%, 0.46–10.6) (31). Coleman et al. also confirmed that hemodynamically significant PDA increases the risk of AKI by almost double that of non-hemodynamic PDAs (1). A meta-analysis showed PDA as one of the risk factors for AKI in premature neonates, with an incidence of 34% (27). Although not significant in this study, the literature suggests that NEC is a strong risk factor for AKI. Bakhoum et al. reported that more than 50% of neonates with NEC developed AKI, suspected to be due to systemic inflammation and hypoperfusion (28).

Most subjects in this study used mechanical ventilation (88.9%) with the median length of ventilator use increasing from 96 h (day 0) to 264 h (day 7), which likely reflects the acuity of our NICU population and local clinical practice. In our unit, preterm neonates with suspected LONS frequently require ventilatory support due to the combined effects of prematurity-related respiratory vulnerability and sepsis-related respiratory failure/hemodynamic instability, and some referrals may arrive already intubated. Bivariate analysis showed that ventilator use was not significant to the incidence of AKI (*p* = 0.603). This high baseline ventilation rate reduces variability and may limit the ability of ventilation status alone to discriminate AKI risk in this cohort. In contrast to previous meta-analysis that showed mechanical ventilation as the main risk factor for AKI in preterm infants, this effect was more clearly seen when large sample counts and patient variations were more heterogeneous (27). A retrospective study stated that the use of mechanical ventilation will increase the risk of AKI by an OR of 9.4 [95% CI, 4 (2.0−43.5)] (29). Most subjects in this study used invasive ventilators; only four infants did not use invasive ventilators, as the variation was low, causing the analysis to be less robust in detecting differences.

In this study, a birth weight of ≤1, 357 g was shown to significantly increase the risk of AKI in preterm neonates with LONS. ROC analysis showed an area under the curve (AUC) of 0.708 (*p* = 0.026) with a sensitivity of 72.2% and a specificity of 77.8%. In the categorical analysis, infants with a birth weight of ≤1, 357 g have an almost three times higher risk of developing AKI compared to infants >1, 357 g (RR, 2.91; 95% CI, 1.31–6.45; *p* = 0.003). In multivariate logistic regression, LBW remained an independent risk factor with an adj OR of 5.33 (95% CI, 0.68–41.45), although the significance was decreased after adjusting for variables. LBW is associated with low nephron endowment, tubule immaturity, vascular and inflammatory susceptibility, and the presence of other comorbidities such as persistent pulmonary hypertension of the newborn, respiratory distress syndrome, and the use of inotropes and ventilators (2, 22, 30).

The RRI variable did not show a significant correlation with the risk of AKI. Even though the median RRI value increased gradually from day 0 (0.78; IQR, 0.74–0.88) to day 7 (0.87; IQR, 0.79–0.93), the comparison between the high- and low-risk AKI groups was not significant at all points of time. ROC analysis also showed that the discriminative ability of RRI was low. These findings indicate that despite an increasing trend in RRI over time, intergroup differences remain inconsistent, and the overall predictive utility of RRI as a stand-alone biomarker for AKI is low. This phenomenon may be attributed to the dynamic regulation of renal perfusion mediated by a balance of renal vascular vasoconstrictors—such as endothelin—and vasodilators, including atrial natriuretic peptide (ANP), bradykinin, kallikrein, prostaglandins, and nitric oxide (32). During the perinatal period, renal efferent arteriolar vasoconstriction is primarily governed by angiotensin II, which is synthesized in substantial amounts owing to angiotensin-converting enzyme production in the lungs and kidneys. Concurrently, levels of renal vasodilators such as ANP and adrenomedullin increase from the first day through the second week of neonatal life, contributing to reduced renal vascular resistance and enhanced renal blood flow—processes that confer renal protection. In pathological states such as sepsis, which elevate renal vascular resistance through hypoxia and impaired perfusion, these endogenous vasodilatory mechanisms may serve to mitigate renal injury (33).

RRI in this study is comparative to the nSOFA score, which is a scoring system developed to assess organ dysfunction in neonates, especially in the context of infection and critical conditions. In this study, nSOFA was shown to be significant on days 0, 1, and 7 for the risk of AKI in neonates with LONS. This is in line with the concept that the nSOFA score reflects the degree of systemic organ dysfunction due to sepsis, which is related to the occurrence of renal perfusion disorders and AKI. Wynn et al. reported that nSOFA significantly differentiated survivors and non-survivors of LONS on ELBW infants, with an AUC reaching 0.93 within 12 h of evaluation (17). A larger multicenter study also showed nSOFA discrimination against mortality with an AUC of 0.88–0.95 (33). Neonatal sepsis mortality is largely caused by AKI-related complications; this supports nSOFA's validity as a mortality predictor and also reinforces its role as an indicator of AKI risk. Recent evidence by Wildes et al. found an incidence of AKI of 48.6% in neonates with LONS and showed that AKI was closely related to mortality (OR 9.4) (34). Although the multivariate analysis by Wildes et al. did not reveal a significant association between the nSOFA score and AKI, these results suggest that elevation of the nSOFA score, indicative of multi-organ dysfunction, increases the susceptibility of neonates to AKI (34).

This study supports the utility of nSOFA as a valuable risk assessment tool for AKI in preterm neonates with LONS particularly when assessed through serial monitoring as shown in bivariate and multivariate analysis. nSOFA scores on days 0 and 1, with cutoff values of >1 and >5, respectively, demonstrated high specificity (83.3% and 100%) but low sensitivity (44.44% and 27.78%), with AUCs of 0.637 and 0.650, while the nSOFA score on day 7 maintained significance in multivariate analysis at a cutoff >3, exhibiting a sensitivity of 61.1%, a specificity of 77.78%, an AUC of 0.724, and a *p*-value of 0.010. Multivariate analysis revealed that the nSOFA score on day 7 was associated with an OR of 10.1 for AKI risk. This indicates that neonates with elevated nSOFA scores on day 7 had over a 10-fold increased likelihood of developing AKI compared to those with lower scores, independent of other variables. These findings highlight that persistent organ dysfunction through the first week of sepsis is a robust predictor of AKI development. This is consistent with existing literature reporting that sepsis-related mortality predicted by nSOFA correlates with AKI complications (34).

This study’s strengths include the application of the novel AKI risk stratification tool, the STARZ score, which enables risk discrimination prior to AKI onset, and the assessment of the nSOFA score and RRI in comparison with the STARZ score. The prospective design and collection of data at multiple time points enhances the diagnostic accuracy. However, limitations comprise its single-center setting, which limits generalizability to the broader neonatal population. The study did not incorporate emerging biomarkers such as NGAL, cystatin C, and KIM-1, among others, precluding direct comparative evaluation of AKI diagnostics. Additionally, data were collected at limited discrete time points, failing to capture continuous daily hemodynamic changes. Although nSOFA, RRI, and STARZ scores were analyzed concurrently, the study has not yet developed a composite prediction model, leaving the full potential of integrating these instruments unexplored. Blood culture positivity could not be evaluated because culture results were not consistently documented/available in the dataset. Consequently, our findings should be interpreted in the context of probable LONS defined by the SPS rather than exclusively culture-confirmed sepsis. Future studies should incorporate standardized microbiological confirmation to examine whether pathogen-proven infection modifies AKI risk and the performance of nSOFA and RRI.

## 5 Conclusion

Neonatal AKI remains a major challenge in the NICU, and earlier recognition is essential to reduce morbidity and mortality. In preterm neonates with probable LONS, our findings suggest that serial nSOFA monitoring—particularly an nSOFA score >3 on day 7—provides clinically useful stratification of high-risk AKI, supporting its role as a practical bedside tool. Although RRI and STARZ increased during the septic course, nSOFA demonstrated stronger discriminatory performance between risk groups in this cohort. Integrating serial organ dysfunction assessment with renal perfusion evaluation may help clinicians identify vulnerable infants earlier and guide closer monitoring and timely supportive interventions. Further multicenter studies with larger samples are needed to validate these cutoffs and define the optimal timing and combination of predictors for routine clinical implementation.

## Data Availability

The raw data supporting the conclusions of this article will be made available by the authors, without undue reservation.
